# Can statistic adjustment of OR minimize the potential confounding bias for meta-analysis of case-control study? A secondary data analysis

**DOI:** 10.1186/s12874-017-0454-x

**Published:** 2017-12-29

**Authors:** Tianyi Liu, Xiaolu Nie, Zehao Wu, Ying Zhang, Guoshuang Feng, Siyu Cai, Yaqi Lv, Xiaoxia Peng

**Affiliations:** 1 Department of Epidemiology and Biostatistics, School of Public Health, Capital Medical University, Beijing, China; 2 Center for Clinical Epidemiology & Evidence-based Medicine, Beijing Children’s Hospital, Capital Medical University, No. 56 Nanlishi Road, Beijing, 100045 China; 3 National Key Discipline of Pediatrics, Ministry of Education, Beijing, China

**Keywords:** Statistic adjustment, Confounding bias, Case-control study, Meta-analysis

## Abstract

**Background:**

Different confounder adjustment strategies were used to estimate odds ratios (ORs) in case-control study, i.e. how many confounders original studies adjusted and what the variables are. This secondary data analysis is aimed to detect whether there are potential biases caused by difference of confounding factor adjustment strategies in case-control study, and whether such bias would impact the summary effect size of meta-analysis.

**Methods:**

We included all meta-analyses that focused on the association between breast cancer and passive smoking among non-smoking women, as well as each original case-control studies included in these meta-analyses. The relative deviations (RDs) of each original study were calculated to detect how magnitude the adjustment would impact the estimation of ORs, compared with crude ORs. At the same time, a scatter diagram was sketched to describe the distribution of adjusted ORs with different number of adjusted confounders.

**Results:**

Substantial inconsistency existed in meta-analysis of case-control studies, which would influence the precision of the summary effect size. First, mixed unadjusted and adjusted ORs were used to combine individual OR in majority of meta-analysis. Second, original studies with different adjustment strategies of confounders were combined, i.e. the number of adjusted confounders and different factors being adjusted in each original study. Third, adjustment did not make the effect size of original studies trend to constringency, which suggested that model fitting might have failed to correct the systematic error caused by confounding.

**Conclusions:**

The heterogeneity of confounder adjustment strategies in case-control studies may lead to further bias for summary effect size in meta-analyses, especially for weak or medium associations so that the direction of causal inference would be even reversed. Therefore, further methodological researches are needed, referring to the assessment of confounder adjustment strategies, as well as how to take this kind of bias into consideration when drawing conclusion based on summary estimation of meta-analyses.

**Electronic supplementary material:**

The online version of this article (10.1186/s12874-017-0454-x) contains supplementary material, which is available to authorized users.

## Background

Meta-analysis is a widely used statistical technique to synthesize results of several independent homogeneous primary studies [1]. When results of different individual studies are conflicted, a well-conducted meta-analysis will help to explore the heterogeneity resource and to improve the precision of effect estimation by increasing sample size of the same research question [2]. The basic assumption of meta-analysis is that each included individual study provides an unbiased estimate of the effect, i.e. the variability of the results of the studies is only attributed to random variation [3]. Meta-analysis method is weighted average of point estimates from each included individual studies, which is based on the standard errors of the estimates [4]. The randomized control trials (RCTs), controlling possible bias by random allocation procedure and blinding, is universally acknowledged as a “combinable” study type for meta-analysis [5].

In recent years, meta-analysis has been widely used in combining estimations of other types of studies, including cross-sectional study, cohort study and case-control study [6]. When there might be no or a few RCTs, synthesizing results from observational studies is needed to generate available evidence [7]. However, the quality of such kind of meta-analysis and the validity of combining heterogeneous effect sizes are suspected [3, 8]. Compared with RCTs, inherent risk of bias in observational study may lead to bias of summary effect size, making the variability of the results among individual studies not simply attributed to randomized variation, i.e. included individual studies do not meet the basic assumption of meta-analysis application [9, 10].

Confounding is a kind of important bias in observational studies, especially in case-control study. The common used confounding control strategies include limiting included subjects, matching important confounding factors in study design, as well as stratification, adjustment and propensity scores in analysis phase. Logistic regression model is widely used to adjust confounders in research practice, which is flexible to control multiple confounders simultaneously.

In theory, overall consideration and adjustment of potential confounders make the estimation of effect size closer to the true effect value better. However, the difference of model building and fitting may lead to ambiguous adjusted results, which make uncertain bias occur in pooling analysis [7, 11]. Although it is well-known that bias indeed exist in meta-analysis of observational studies, but it is still unclear that how the different adjusting strategies in case-control studies influent the summary effect size of meta-analysis. This secondary data analysis focused confounder adjustment strategies in case-control study, i.e. how many confounders that original studies adjusted and what the variables are, as well as whether statistical adjustment of ORs would minimize the potential confounding bias for meta-analysis of case-control study.

## Methods

### Identifying topic of the case

Our aim is to explore possible bias caused by confounder adjustment strategy in case-control study, i.e. how individual studies deal with confounders and how the difference of strategies would impact the summarized effect size of meta-analysis. As an example, the studies focused on the association between breast cancer and passive smoking among non-smoking women, in which ORs varies from 1.5 to 2.0. The reasons behind why we selected this example include: (1) numbers of relevant case-control studies which had been conducted in different context (areas, populations, published years); (2) the existed results shown that there was indeed positive association between passive smoking exposure and breast cancer development [12]; (3) the weaker or medium strength of association, which is more susceptible compared with the strong association.

### Search strategy

Meta-analyses focused on passive smoking and breast cancer were identified through three English databases (MEDLINE, EMbase and Cochrane Library) and three Chinese databases (CNKI, WanFang, and VIP), using terms “passive smoking”, or “tobacco”, or “environmental tobacco smoke or its’ abbreviation of ETS”, combined with “breast neoplasms”, or “breast cancer” and “meta-analysis”, or “systematic review”. The search strategy was attached in Additional file 1.

### Eligibility

We included all meta-analyses of associations between passive smoking and breast cancer among non-smoking women or meta-analyses in which the results of a passive smoking among non-smoking women subgroups were able to be obtained. Meta-analyses published between January 1966 and December 2016 were included. In addition, we excluded duplications, meeting abstractions, and meta-analyses which we couldn’t obtain full-text from.

### Assessment of methodological quality

We used the *A measurement tool to assess systematic reviews (AMSTAR)* to evaluate the risk of methodological quality of meta-analyses which were included in the present study [13]. The assessment of methodological quality was conducted by two authors (Wu ZH, and Zhang Y) independently and disagreements were adjudicated by the third researcher (Liu TY).

### Data extraction

All full-texts of targeted meta-analyses were screened. At the same time, all original articles including interested subgroups were extracted. At the level of meta-analysis, we extracted ID, author, year of publication, country, number of included study, summarized OR and its’ 95% CI (Confidence Interval), result of heterogeneity test, selection of crude/adjusted OR to conduct pooling analysis. At the original study level, we extracted author, year of publication, type of study, sample size, crude or adjusted OR and its’ 95% CI, number of confounder adjusted and the variable of confounders. Data extraction was conducted by two researchers independently (Zhang Y and Wu ZH). Any disagreement in study selection and data extraction was adjudicated by the third researcher (Liu TY).

### Data analysis

We calculated the relative deviation (RD), i.e. crude OR minus adjusted OR, then divided by crude OR in each original study to detect how magnitude would the adjustment impact the effect size. We also listed how many confounders each original study adjusted, as well as what the variables were. To describe the distribution of adjusted OR with different number of adjusted confounders, a scatter diagram was sketched using adjusted OR of original studies as Y-axis and using the number of adjusted variables as X-axis. The data management was conducted using Microsoft Excel 2010. The scatter diagram was performed under software of SAS 9.4.

## Results

### Study characteristics

**Fig. 1:**
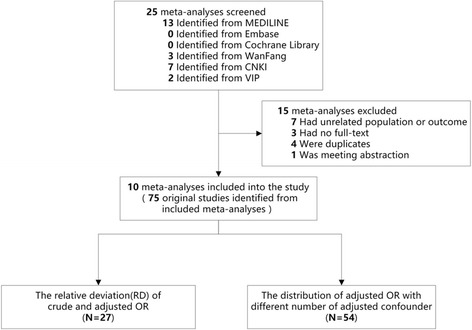
Flow diagram of included studies

Totally 10 meta-analyses were included in this study. From the 10 meta-analyses, the association between passive smoking with breast cancer risk for non-smoking women is the primary outcomes, while the association between passive smoking with breast cancer risk for non-smoking women was reported as subgroup analysis in other 6 meta-analyses (Fig. 1). All interested details of eligible meta-analyses were presented in Table 1 [14–23], and the item by item AMSTAR assessment score of each included meta-analyses were shown in Additional file 2. Summarized ORs vary from 1.21 to 1.94. The results of heterogeneity tests showed that there existed heterogeneous in 8 of 10 meta-analyses.

**Table 1:** Summary of basic characteristics of 10 included meta-analyses

Study	Year	Country	Summary OR	95% CI		Test of heterogeneity	Crude or adjusted OR to combined^a^	AMSTAR score
				Lower	Upper			
Lee PN [14]	2016	UK	1.22	1.09	1.37	No exact value, *P* < 0.001	Mixed OR	6
Chen Z [17]	2015	China	1.67	1.27	2.21	χ^2^ = 64.71, *P* < 0.00001	Mixed OR	9
Macacu A [15]	2015	France	1.30	1.10	1.54	χ^2^ = 91.85, *P* < 0.0001	Mixed OR	8
Chen C [16]	2014	China	1.54	1.35	1.73	I^2^ = 7.9%, *P* = 0.367	All adjusted OR	9
Ma J [18]	2011	China	1.23	1.12	1.40	χ^2^ = 32.68, *P* = 0.000	Mixed OR	6
Pirie K [19]	2008	UK	1.21	1.11	1.32	χ^2^ = 13.8, *P* = 0.0002	All adjusted OR	6
Sadri G [20]	2007	Iran	1.38	1.16	1.65	χ^2^ = 5.83, *P* = 0.32	All adjusted OR	6
Zhou XB [21]	2006	China	1.94	1.80	2.10	χ^2^ = 20.13, *P* < 0.01	All crude OR	7
Johnson KC [22]	2005	Canada	1.90	1.53	2.37	No exact value, *P* < 0.05	Mixed OR	6
Khuder SA [23]	2001	USA	1.41	1.14	1.75	χ^2^ = 34.6, P < 0.01	Mixed OR	7

All crude OR: using only crude OR of original studies to combining summary effect size

All adjusted OR: using only adjusted OR of original studies to combining summary effect size

Mixed OR: using mixed crude OR and adjusted OR of original studies to combining summary effect size

^a^ *CI* confidence interval, *AMSTAR* A measurement tool to assess systematic reviews

Ten included meta-analyses deal with OR in various ways, including all crude OR, all adjusted OR, and mixed crude/ adjusted OR of original studies when combining effect size. All of crude ORs in original studies were pooled in only 1 meta-analysis [21]. Three meta-analyses [16, 19, 20] summarized all adjusted OR of original studies. Other 6 meta-analyses [14, 15, 17, 18, 22, 23] combined effect size using mixed crude OR and adjusted OR of original studies.

After removing the overlap of original studies included in 10 meta-analyses, there were totally 75 original studies that were re-analyzed, including 11 prospective studies and 64 retrospective studies (listed in Additional file 3). Totally, we obtained 112 estimations of ORs from 10 included meta-analyses, 24 ORs of which were included repeatedly in different meta-analyses, i.e. 88 results were non-repetition included in different meta-analyses. The reason why the number of non-repetition result (88 ORs) was bigger than 75 was that there were several estimations of OR in some original studies.

### The relative deviation (RD) of crude and adjusted OR in original study

**Fig. 2:**
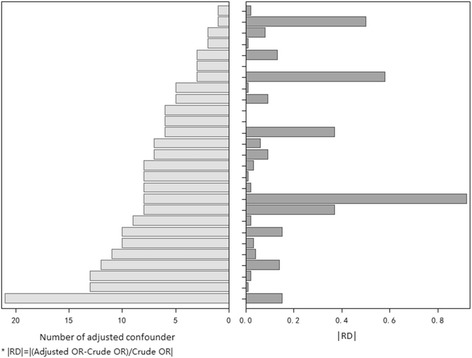
The relative deviation (RD) of crude adjusted OR (*N* = 27)

In 64 original retrospective studies, full-text of 15 studies could not be obtained, and 16 studies did not provide fourfold table of case and control group so that the crude OR were unknown, 6 studies did not clarify the adjustment strategy. Therefore, we got finally 27 studies to analyze the RD of crude and adjusted OR in original studies (Fig. 2). Figure 2 showed RDs between crude and adjusted OR vary from 0.00 to 0.92. Furthermore, there were no obviously association between number of adjusted variable and the |RD| so that it is hard to compare the effect of adjustment. In addition, the RD and the confounding adjustment strategy of each original study were listed in Additional file 4.

### The distribution of adjusted OR with different number of adjusted confounder

**Fig. 3:**
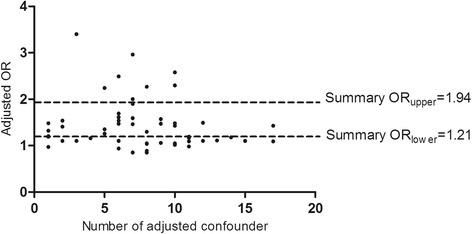
The distribution of adjusted OR with different number of adjusted confounder (*N* = 54)

In 88 non-repetition OR estimations from 75 original studies, there were 23 crude ORs, and 9 crude ORs from case-control studies conducted by 1:1 matching without statistical adjustment, and 2 studies did not clarify the number of adjusted confounding variables. As a result, 54 adjusted ORs were scattered by the number of adjusted confounders (Fig. 3). There was no obvious trend of constringency with the increasing of the adjusted confounders in Fig. 3, suggesting that statistical adjustment may fail to reduce the systematic error caused by confounding bias.

## Discussion

In summary, we detected potential inconsistency in meta-analysis of case-control studies, which may influence the precision of the summarized effect size. First, mixed unadjusted and adjusted OR were used to combine effect size in certain meta-analysis. In addition, the RD of crude and adjusted OR was quite variable, and there was insufficient evidence to adjudicate which estimation are more precise. Second, original studies with different adjustment strategies for confounders were combined, i.e. the number of adjusted factors and different factors for adjustment in each original study. Third, confounder adjustment did not make the effect size of individual studies trend to constringency, suggesting that model fitting might have failed to control the systematic error caused by confounding. All the issues above may have lead to a wide range of pooled effect size from different meta-analysis (1.21–1.94), which may introduce potential bias into meta-analysis, especially failing to improve the precision of effect size for weaker or medium association. The heterogeneity among original studies could be made complicated when different adjusting strategies are extremely variability so that the synthesis of evidences would become controversial [24].

It is well known that there are two kinds errors that impact the causal inference; the random error and the systematic error. Random error impact precision of the estimate, whist systematic error influents accuracy. Increasing the sample size will contribute mainly to reduce the random error, but has no effect on systematic error. To reduce the bias leaded by systematic error, researchers should consider and assess the significance of potential bias since study design phase. The inherent risk of bias in case-control study (i.e. selection bias, information bias and confounding bias) could make the accuracy of estimation suspicious. In general, strategies for preventing selection bias and information bias are considered at the stage of research design and the implementation stage. Statistical strategies are commonly used to adjust confounding bias in data analysis stages. In theory, when selection bias and information bias have been well-controlled in the stages of study design and implementation, overall consideration and adjustment of potential confounders in data analyses stage could better the estimation of effect size close to the true effect size. Therefore, the adjusted OR of individual studies should tend to be constringency after the systematic error reduced validly. However, the results in this study have shown that various adjustment models have no obvious effect.

In fact, it is difficult to adjudicate whether adjusted ORs tend to more validate estimation of OR by adjustment strategies for confounders because both of design and statistical analysis impact the adjusted results. Results of adjustment using multiple regression greatly depend on whether each confounder was measured, how precisely measurement was, and how each variable was taken into the regression model [25]. In addition, residual confounding of statistical model may lead to unpredictable effect, which can not be neglected [26]. The validity of study is not only statistical consideration, more importantly, relating to research design at the protocol stage, especially for case-control study which is susceptible for bias [27].

The reason why we selected the association between breast cancer and passive smoking among non-smoking women as the case is that the association is weak or medium because weak or medium association is more likely impacted by potential bias, even could be reversed the direction of estimation. For example, one of included meta-analysis (Peter 2016) conducted subgroup analyses by number of confounders and found there was significant heterogeneity between different adjustment strategy groups (adjusted for nine or more confounders, random-effects OR of 1.05(0.93–1.19) compared with eight or less confounder, random-effects OR 1.23(1.03–1.45)) [14], which suggested that the different adjustment strategies would lead to the opposite conclusion.

On the other hand, we usually assess the quality of each included original studies using *The Newcastle-Ottawa Scale (NOS)*, which take three broads of bias into consideration roundly, i.e. the selection of the study groups; the comparability of the groups, and the ascertainment of either the exposure or outcome when conducting a meta-analysis for observational studies [28]. However, NOS doesn’t involve how to detect important confounders, how to measure and control them. What’s more, there has no uniform recommendation regarding the selection of crude OR or adjusted OR in meta-analyses, as well as how to consider the effects of statistical adjustment for confounding variables. There is also little recommendation on the assessment of confounder adjustment strategies and how to take the potential bias introduced by different adjustment strategies among individual studies into consideration when we intend to make summarized estimation of effect size by meta-analysis of case-control studies.

Although we detected several existing inconformity in meta-analysis of case-control study based on a case of passive smoking and breast cancer, the limitation of the present study, as a case of secondary data analysis is needed to make further consideration. The impacts of different confounding adjustment strategies on weak or medium association in case-control studies couldn’t be generalized all the meta-analyses of case-control studies. Another limitation is that it was not clear that the true effect size of the association between breast cancer and passive smoking among non-smoking women so that we couldn’t assess exactly how the different adjustment strategies impact the effect size of each original study. In addition, individual patient data meta-analysis (IPD) should benefit to clarify the impact of confounding adjustment strategy on better precisely estimations, however, we failed to ask researchers of original studies for their primary data. Even though, the findings in the present study provided a new profile to detect potential bias sources in meta-analysis of case-control studies.

The results of the study shows that the difference of adjustment strategies of confounding factors in case-control studies may lead to bias of summary effect size in meta-analysis. Especially when the association strength is weak or medium, such bias will even reverse the direction of causal inference. However, whether it is reasonable to combine mixed unadjusted and adjusted estimates of original study in meta-analyses? How much deviation is caused by confounder adjustment strategy of individual study in meta-analysis of case-control study? How should researchers deal with the heterogeneity introduced by different confounder adjustment strategy? In order to address the above questions, further simulation study based on different adjustment strategy scenarios can provide more robust evidence, while the findings in present study provided useful recommendations for simulation design to make the simulation scenarios reflect the reality case-control studies situation in a better way.

## Conclusions

The heterogeneity of confounder adjustment strategies in case-control studies may lead to further bias for summary effect size in meta-analyses, especially for weak or medium association so that the direction of causal inference would be even reversed. Therefore, further methodological research is needed, referring to the assessment of confounder adjustment strategies, as well as how to take this kind of bias into account when drawing conclusion based on summary estimation of meta-analyses.

## Additional files

Additional file 1: Search strategy. (PDF 86 kb)

Additional file 2: AMSTAR score of included meta-analysis. (PDF 87 kb)

Additional file 3: Summary of included original study. (PDF 1 mb)

Additional file 4: Summary of relative deviation and the confounding adjustment strategy of each original study. (PDF 709 kb)

## Abbreviations

AMSTAR: A measurement tool to assess systematic reviews
CI: Confidence Interval
NOS: The Newcastle-Ottawa Scale
RCTs: Randomized control trials
RD: Relative deviation
