# Supplementary material for: Can statistic adjustment of OR minimize the potential confounding bias for meta-analysis of case-control study? A secondary data analysis
Source: BMC Med Res Methodol. 2017 Dec 29;17:179. doi: 10.1186/s12874-017-0454-x (PMC5747180; doi:10.1186/s12874-017-0454-x)
Supplement: Supplementary file 1 — Search strategy. (PDF 86 kb) [file 12874_2017_454_MOESM1_ESM.pdf]

Additional file 1: Search strategy.

Search	Query
#1	passive[Title/Abstract]
#2	smok*[Title/Abstract]
#3	tobacco[Title/Abstract]
#4	"environmental tobacco smoke"[Title/Abstract]
#5	ETS[Title/Abstract]
#6	(passive[Title/Abstract]) AND smok*[Title/Abstract]
#7	(passive[Title/Abstract]) AND tobacco[Title/Abstract]
#8	((("environmental tobacco smoke"[Title/Abstract]) OR ETS[Title/Abstract]) OR ((passive[Title/Abstract]) AND smok*[Title/Abstract])) OR ((passive[Title/Abstract]) AND tobacco[Title/Abstract])
#9	"breast neoplasms"[MeSH Terms]
#10	"breast cancer"[Title/Abstract]
#11	("breast neoplasms"[MeSH Terms]) OR "breast cancer"[Title/Abstract]
#12	"meta-analysis"[Title/Abstract]
#13	"meta-analysis"[Publication Type]
#14	"meta-analysis"[MeSH Terms]
#15	(("meta-analysis"[Title/Abstract]) OR "meta-analysis"[Publication Type]) OR "meta-analysis"[MeSH Terms]
#16	overview[Title/Abstract]
#17	(((("meta-analysis"[Title/Abstract]) OR "meta-analysis"[Publication Type]) OR "meta-analysis"[MeSH Terms])) OR overview[Title/Abstract]
#18	((((((("meta-analysis"[Title/Abstract]) OR "meta-analysis"[Publication Type]) OR "meta-analysis"[MeSH Terms])) OR overview[Title/Abstract])) AND (("breast neoplasms"[MeSH Terms]) OR "breast cancer"[Title/Abstract])) AND (((("environmental tobacco smoke"[Title/Abstract]) OR ETS[Title/Abstract]) OR ((passive[Title/Abstract]) AND smok*[Title/Abstract])) OR ((passive[Title/Abstract]) AND tobacco[Title/Abstract]))
