# Supplementary material for: Can statistic adjustment of OR minimize the potential confounding bias for meta-analysis of case-control study? A secondary data analysis
Source: BMC Med Res Methodol. 2017 Dec 29;17:179. doi: 10.1186/s12874-017-0454-x (PMC5747180; doi:10.1186/s12874-017-0454-x)
Supplement: Supplementary file 2 — AMSTAR score of included meta-analysis. (PDF 87 kb) [file 12874_2017_454_MOESM2_ESM.pdf]

Additional file 2: AMSTAR score of included meta-analyses.

Study	Item 1	Item 2	Item 3	Item 4	Item 5	Item 6	Item 7	Item 8	Item 9	Item 10	Item 11	Total
Alina M, 2015	3	1	1	2	1	1	2	1	1	1	1	8
Chen C, 2014	3	1	1	2	1	1	1	1	1	1	1	9
Chen ZH, 2013	3	1	1	2	1	1	1	1	1	1	1	9
Ma J, 2011	3	3	1	1	1	1	2	1	1	3	2	6
Kirstin Pirie, 2008	1	2	1	2	1	1	2	1	2	2	1	6
Peter NL, 2006	3	3	2	2	1	1	1	1	1	1	3	6
Sadri G, 2006	3	1	1	2	1	1	2	1	1	2	3	6
Johnson KC, 2005	3	3	2	1	1	1	2	1	1	1	3	6
Zhou JB, 2005	1	2	2	2	1	1	1	1	1	1	3	7
Khuder SA, 2001	3	1	1	2	1	1	2	1	1	1	3	7

* AMSTAR: A measurement tool to assess systematic reviews.
