# Supplementary material for: Can statistic adjustment of OR minimize the potential confounding bias for meta-analysis of case-control study? A secondary data analysis
Source: BMC Med Res Methodol. 2017 Dec 29;17:179. doi: 10.1186/s12874-017-0454-x (PMC5747180; doi:10.1186/s12874-017-0454-x)
Supplement: Supplementary file 3 — Summary of included original study. (PDF 1 mb) [file 12874_2017_454_MOESM3_ESM.pdf]

Additional file 3: Summary of included original study (N=75).

1. Ahern TP, Lash TL, Egan KM, Baron JA. Lifetime tobacco smoke exposure and breast cancer incidence. Cancer Causes Control 2009; 20:1837-1844. doi: 10.1007/s10552-009-9376-1.
2. Alberg AJ, Daudt A, Huang HY, et al. N-acetyltransferase 2 (NAT2) genotypes, cigarette smoking, and the risk of breast cancer. Cancer Detect Prev 2004; 28:187-193. doi: 10.1016/j.cdp.2004.04.001.
3. Bonner MR, Nie J, Han D, et al. Secondhand smoke exposure in early life and the risk of breast cancer among never smokers (United States). Cancer Causes Control 2005; 16:683-689. doi: 10.1007/s10552-005-1906-x.
4. Cao KJ, Wu L, Ma GS, et al. Case-control study for breast cancer risk factor in Guangzhou. Zhong Guo Zhong Liu 2001; 10(12):702-4. [In Chinese].
5. Chilian-Herrera OL, Cantor KP, Herna ´ndez-Ramı ´rez R, Lo ´pez-Carrillo L. Passive smoking increases the risk of breast cancer among pre- and post-menopausal Mexican women (Abstract). Cancer Epidemiol Biomarkers Prev 2010; 19(10 Suppl 1):A99.
6. Conlon MSC, Johnson KC, Bewick MA, et al. Smoking (active and passive), N-acetyltransferase 2, and risk of breast cancer. Cancer Epidemiol. 2010; 34:142–9.
7. De Silva M, Senarath U, Gunatilake M, Lokuhetty D. Prolonged breastfeeding reduces risk of breast cancer in Sri Lankan women: a case-control study. Cancer Epidemiol. 2010; 34:267–73.
8. Dossus L, Boutron-Ruault M-C, Kaaks R, et al. Active and passive cigarette smoking and breast cancer risk: results from the EPIC cohort. Int J Cancer. 2014; 134:1871–88.
9. Friedenreich CM, Bryant HE, Alexander F, Hugh J, Danyluk J, Page DL. Risk factors for benign breast biopsies: a nested case-control study in the Alberta breast screening program. Cancer Detect Prev. 2001; 25: 280-291.
10. Gammon MD, Eng SM, Teitelbaum SL, et al. Environmental tobacco smoke and breast cancer incidence. Environ Res 2004; 96:176-185. doi:

10.1016/j.envres.2003.08.009.

11. Gao CM, Ding JH, Li SP, et al. Active and passive smoking, and alcohol drinking and breast cancer risk in Chinese women. APJCP, 2013; 14: 993-996.
12. Hanaoka T, Yamamoto S, Sobue T, et al. Active and passive smoking and breast cancer risk in middle-aged Japanese women. Int J Cancer. 2005; 114: 317–22.
13. Hirayama T. Cancer mortality in nonsmoking women with smoking husbands based on a large-scale cohort study in Japan. Prev Med. 1984; 13: 680–90.
14. Hsieh YC, Lee CH, Tu SH, et al. CHRNA9 polymorphisms and smoking exposure synergize to increase the risk of breast cancer in Taiwan. Carcinogenesis 2014; 35:2520-2525. doi: 10.1093/carcin/bgu179.
15. Hu M, Han D, Sun S, et al. Bleomycin-induced mutagen sensitivity, passive smoking, and risk of breast cancer in Chinese women: a case-control study. Cancer Causes Control 2013; 24:629-636. doi: 10.1007/s10552-012-0137-1.
16. Ilic M, Vlajinac H, Marinkovic J. Cigarette Smoking and Breast Cancer: a Case-control Study in Serbia. Asian Pacific Journal of Cancer Prevention. 2013; 14:6643-6647. doi: 10.7314/apjcp.2013.14.11.6643.
17. Jee SH, Ohrr H, Kim IS. Effects of husbands' smoking on the incidence of lung cancer in Korean women. Int J Epidemiol. 1999; 28: 824–28.
18. Jia YP. Case-control study for female breast cancer. Shi Yong Yi Xue Za Zhi. 2001. 17(7):646. [in Chinese].
19. Jianchen Z, Zhenbin X, Shoufeng Z, et al. Match case-control study for detecting risk factors of breast cancer in women living in Xinxiang, Henan province. Henan J Oncol. 2003. 16: 201-203.
20. Johnson KC, Hu J, Mao Y, Canadian Cancer Registries Epidemiology Research G. Passive and active smoking and breast cancer risk in Canada, 1994-97. Cancer Causes Control. 2000; 11:211-221.
21. Johnson KC, Hu J, Mao Y. Passive and active smoking and breast cancer risk in Canada, 1994–97. The Canadian Cancer Registries Epidemiology Research Group. Cancer Causes Control. 2000; 11: 211–21.
22. Johnson KR, semenciw R, Hu J, Mao Y. Active and passive smoking and

pre-menopausal breast cancer. Am J Epidemiol. 1998; 147:S68, 272.

23. Ju L, Liu HR, Song PD, et al. Case-control study for risk of breast cancer in college female. Zhong Guo Xiao Yi. 2001; 15(2):94. [In Chinese].
24. Jun L, Hua L, Su-ying R, et al. A case-control study on environmental risk factors of female breast cancer in Tangshan city. Chin J Public Health. 2007; 23: 312-314.
25. Kropp S, Chang-Claude J. Active and passive smoking and risk of breast cancer by age 50 years among German women. Am J Epidemiol. 2002. 156: 616–26.
26. Lash TL, Aschengrau A. A null association between active or passive smoking and breast cancer risk. Breast Cancer Res Treat. 2002. 75: 181–84.
27. Lash TL, Aschengrau A. Active and passive cigarette smoking and the occurrence of breast cancer. Am J Epidemiol. 1999. 149: 5-12.
28. Li J, Liu H, Rong S, et al. [A case-control study on environmental risk factors of female breast cancer in Tangshan]. Zhong Guo Gong Gong Wei Sheng. 2007. 23:312-314.
29. Liang Z, Tianfeng H, Yali J, et al. A case-control study on risk factors of breast cancer. Chian Cancer. 2009; 18:27-30.
30. Lin J, Yu J. [A case-control study on risk factors of breast cancer among women in Cixi]. Zheijiang Yu Fang Yi Xue, 2008; 20:3-5.
31. Lin XY, Xu GF, Xu H, et al. [Case-control study of breast cancer risk in Jinan]. Shan Dong Da Xue Xiao Bao 2001; 39(6):552-3.
32. Lissowska J, Brinton LA, Zatonski W, et al. Tobacco smoking, NAT2 acetylation genotype and breast cancer risk. Int J Cancer. 2006. 119:1961-1969. doi: 10.1002/ijc.22044.
33. Liu L, Wu K, Lin X, et al. Passive smoking and other factors at different periods of life and breast cancer risk in Chinese women who have never smoked—a case-control study in Chongqing, People’s Republic of China. APJCP. 2000.1:131-137.
34. Liu LJ, Wu KN, Lin XN, et al. [Case-control study of breast cancer risk factor of female in Chongqing]. Zhong Guo Zhong Liu. 1998. 7(11):12-4.

35. Liu LJ, Wu KN, Wang JH, et al. The relationship between early life experience and risk for breast cancer in premenopausal women. Eur J Cancer. 1996. 32: 28-29.
36. Liu RX, Huang WM, Guo GN. [Logistic regression of female breast cancer risk factor. Zhong Liu Fang Zhi Yan Jiu. 1994. 21(2):102-4].
37. Luo J, Margolis KL, Wactawski-Wende J, et al. Association of active and passive smoking with risk of breast cancer among postmenopausal women: a prospective cohort study. BMJ. 2011; 342:d1016.
38. Marcus PM, Newman B, Millikan RC, et al. The associations of adolescent cigarette smoking, alcoholic beverage consumption, environmental tobacco smoke, and ionizing radiation with subsequent breast cancer risk (United States). Cancer Cause Control. 2000. 11:271-278.
39. Mechanic LE, Millikan RC, Player J, et al. Polymorphisms in nucleotide excision repair genes, smoking and breast cancer in African Americans and whites: a population-based case-control study. Carcinogenesis. 2006. 27:1377-1385. doi: 10.1093/carcin/bgi330.
40. Millikan RC, Pittman GS, Newman B et al. Cigarette smoking, N-acetyltransferases 1 and 2, and breast cancer risk. Cancer Epidemiol Biomarkers Prev. 1998. 7: 371–78.
41. Morabia A, Bernstein M, Heritier S, et al. Relation of breast cancer with passive and active exposure to tobacco smoke. Am J Epidemiol. 1996. 143:918-928.
42. Morabia A, Bernstein M, Heritier S, Khatchatrian N. Relation of breast cancer with passive and active exposure to tobacco smoke. Am J Epidemiol. 1996.143: 918–28.
43. Morabia A, Bernstein MS, Bouchardy I, Kurtz J, Morris MA. Breast cancer and active and passive smoking: the role of the N-acetyltransferase 2 genotype. Am J Epidemiol. 2000. 152: 226-232.
44. Nishino Y, Minami Y, Kawai M, et al. Cigarette smoking and breast cancer risk in relation to joint estrogen and progesterone receptor status: a case-control study in Japan. Springerplus. 2014. 3:65. doi: 10.1186/2193-1801-3-65.

45. Pimhanam C, Sangrajrang S, Ekpanyaskul C. Tobacco Smoke Exposure and Breast Cancer Risk in Thai Urban Females. Asian Pacific Journal of Cancer Prevention. 2014. 15:7407-7411. doi: 10.7314/apjcp.2014.15.17.7407.
46. Pirie K, Beral V, Peto R, et al. [Passive smoking and breast cancer in never smokers: prospective study and meta-analysis. Int J Epidemiol. 2008. 37:1069–79.
47. Ren X. A 1:1 case-control study on risk factors of breast cancer]. Dalian, Liaoning, China: Dalian Medical University. 2008.
48. Roddam AW, Pirie K, Pike MC et al. Active and passive smoking and the risk of breast cancer in women aged 36-45 years: a population based case-control study in the UK. Br J Cancer. 2007. 97: 434–39.
49. Rollison DE, Brownson RC, Hathcock HL, et al. Case-control study of tobacco smoke exposure and breast cancer risk in Delaware. BMC Cancer. 2008. 8:157. doi: 10.1186/1471-2407-8-157.
50. Rookus MA, Verloop J, de Vries F, et al. Passive and active smoking and the risk of breast cancer (Abstract (SER)). Am J Epidemiol. 2000. 151:S28.
51. Sagiv SK, Gaudet MM, Eng SM et al. Active and passive cigarette smoke and breast cancer survival. Ann Epidemiol. 2007. 17:385–93.
52. Sandler DP, Everson RB, Wilcox AJ. Passive smoking in adulthood and cancer risk. Am J Epidemiol. 1985. 121: 37–48.
53. Shannon J, Ray R, Wu C, et al. Food and botanical groupings and risk of breast cancer: a case-control study in Shanghai, China. Cancer Epidemiol Biomarkers Prev. 2005. 14:81-90.
54. Shi P, Xu M, Qian Y, et al. [Matched case—control study for detecting risk factors of breast cancer in women living in Wuxi]. Xian Dai Yi Xue Za Zhi. 2010. 37: 2428-2431.
55. Shrubsole MJ, Gao YT, Dai Q, et al. Passive smoking and breast cancer risk among non-smoking Chinese women. Int J Cancer. 2004. 110:605-609. doi: 10.1002/ijc.20168.
56. Sillanpaa P, Hirvonen A, Kataja V, et al. NAT2 slow acetylator genotype as an

important modifier of breast cancer risk. Int J Cancer. 2005. 114:579-584. doi: 10.1002/ijc.20677.

57. Slattery ML, Curtin K, Giuliano AR, et al. Active and passive smoking, IL6, ESR1, and breast cancer risk. Breast Cancer Res Treat. 2008. 109:101–11.
58. Smith SJ, Deacon JM, Chilvers CE. Alcohol, smoking, passive smoking and caffeine in relation to breast cancer risk in young women. UK National Case-Control Study Group. Br J Cancer. 1994. 70: 112–9.
59. Tan H, Liu A, Wang J, et al. [A case control study on multiple risk factors of female breast cancer]. Hunan Yi Ke Da Xue Xue Bao. 1998. 23:38-41.
60. Tang LY, Chen LJ, Qi ML, et al. Effects of passive smoking on breast cancer risk in pre/post-menopausal women as modified by polymorphisms of PARP1 and ESR1. Gene. 2013. 524:84-89. doi: 10.1016/j.gene.2013.04.064.
61. Tong JH, Li Z, Shi J, et al. Passive smoking exposure from partners as a risk factor for ER+/PR+ double positive breast cancer in never-smoking Chinese urban women: a hospital-based matched case control study. PLoS One. 2014. 9:e97498. doi: 10.1371/journal.pone.0097498.
62. Wang P, Xie S, Zhang C, et al. [Relationship of passive smoking and N-acetyltransferase 2 polymorphisms with breast cancer susceptibility]. Zhong Guo Xian Dai Yi Xue Za Zhi. 2011. 21: 3271- 3275.
63. Wang X, Chen W, Lei T, et al. [A case-control study on risk factors of female breast cancer in rural area of Cixi city, Zhejiang province]. Zhong Guo Zhong Liu. 2006. 15: 294-297.
64. Wang Y. [Case-control study on the risk factors of breast cancer based on a cohort population in Jiashan country]. Hangzhou, Zhejiang, China: Zhejiang University; 2006. [In Chinese].
65. Warenberg D, Calle EE, Thun MJ, et al. Passive smoking exposure and female breast cancer mortality. J Natl Cancer Inst. 2000. 92: 1666-1673.
66. Yingqing W. Case-control study on the risk factors of breast cancer in Jiashan county [Unpublished dissertation]. Zhejiang, China: Zhejiang University. 2007.
67. Young E, Leatherdale S, Sloan M, et al. Age of smoking initiation and risk of

breast cancer in a sample of Ontario women. Tob Induc Dis. 2009. 5:4.

68. Yu SC, Wang TJ, Zhang B. Case-control study of female breast cancer in Shenyang. Zhong Guo Gong Gong Wei Sheng Xue Bao. 1991. 10(1):7-9. [In Chinese].
69. Yuanping Z. A case-control study of risk factors of breast cancer. J Pract Med. 2001.17: 646.
70. Zang S, Zhang Y. [Analysis of 312 cases of breast cancer risk factors]. Shi Yong Yi Yao Za Zhi. 2011. 1078-1079.
71. Zha Y. [Case-control study on the risk factors of female breast cancer]. Shi Yong Yi Xue Za Zhi. 2001. 646.
72. Zhang CX, Ho SC, Chen YM, et al. Meat and egg consumption and risk of breast cancer among Chinese women. Cancer Causes Control. 2009. 20:1845-1853. doi: 10.1007/s10552-009-9377-0.
73. Zhao Y, Shi Z, Liu L. [Matched case-control study for detecting risk factors of breast cancer in women living in Chengdu]. Zhonghua Liu Xing Bing Xue Za Zhi. 1999. 20: 91–4.
74. Zhu HH, Gao YT, Blair A, et al. Secondhand smoke and breast cancer risk: a community-based prospective cohort study (Abstract (SER)). Am J Epidemiol. 2006. 163:S98.
75. Zhu M, Tong J, Zhang ZY, et al. [Analysis of breast cancer risk factor]. Zhong Guo Gong Gong Wei Sheng. 2000. 16(3):251.
