# Supplementary material for: Can statistic adjustment of OR minimize the potential confounding bias for meta-analysis of case-control study? A secondary data analysis
Source: BMC Med Res Methodol. 2017 Dec 29;17:179. doi: 10.1186/s12874-017-0454-x (PMC5747180; doi:10.1186/s12874-017-0454-x)
Supplement: Supplementary file 4 — Summary of relative deviation and the confounding adjustment strategy of each original study. (PDF 709 kb) [file 12874_2017_454_MOESM4_ESM.pdf]

Additional file 4: Summary of relative deviation and the confounding adjustment strategy of each original study.

Study	Crude OR	Adjusted OR	Deviation	Relative deviation	Number of adjusted	Variable of adjusted
Alberg AJ, 2004	1.13	1.70	0.57	0.50	1	Age
Conlon et al, 2010	1.13	1.15	0.02	0.02	1	Age
Hirose K, 1995	1.15	1.24	0.09	0.08	2	Age, first-visit year
Roddam AW, 2007	0.90	0.89	-0.01	-0.01	2	Parity (nulliparous, parous) and oral contraceptive use (never user, last use within 5 years, last use 5+ years)
Morabia A, 2000	5.23	5.90	0.67	0.13	3	Age, education, FHBC
Hsieh, 2014	3.40	3.40	0.00	0.00	3	Age, lifetime estrogen exposure, BMI
Friedenreich, 2001	0.89	1.41	0.52	0.58	3	Age, race, sampling fractions
Hu M, 2013	1.69	1.54	-0.15	-0.09	5	Age, physical activity, menopausal status, alcohol, BMI, family history of female cancer
Liang Z, 2009	2.25	2.22	-0.03	-0.01	5	Age, education, age at menarche, family history of female cancer, history of benign breast diseases
Ahern TP, 2009	1.20	1.20	0.00	0.00	6	Age, BMI, menopausal status, parity, alcohol use, FHBC
Mechanic LE, 2006	1.02	1.40	0.38	0.37	6	Age, age at menarche, age at first full-term pregnancy /parity composite, family history and alcohol
Gao, 2013	1.47	1.47	0.00	0.00	6	Age, menopausal status, educational status, occupation, BMI, income
Sillanpaa P, 2005	0.78	0.85	0.07	0.09	7	Age, age at menarche, age at 1st full-term pregnancy, number of pregnancies, 1st-degree family history of breast cancer, history of benign breast diseases, use of alcohol
Tong JH, 2014	1.55	1.46	-0.09	-0.06	7	Age at interview, age at menarche, menopausal status, oral contraceptive use, family history of cancer, alcohol consumption, BMI
Gammon MD, 2004	1.06	1.04	-0.02	-0.02	8	Age, BBD, BMI at age 20, FHBC, history of fertility problems, number of pregnancies, menopausal status, weight in year prior to reference date
Kropp S Chang-Claude, 2002	1.62	1.61	-0.01	-0.01	8	Age, education, region, age at menopause, FHBC, lactation, BMI, alcohol
Lash TL, Aschengrau A, 1999	1.04	2.00	0.96	0.92	8	Age, history of radiation therapy, BMI, history of mother or sister with breast cancer, history of breast cancer, parity, and history of benign breast disease
Millikan RC, 1998	0.95	1.30	0.35	0.37	8	Age, race, age at menarche, age at first full-term pregnancy, parity, FHBC, benign breast biopsy, and alcohol consumption.
Morabia A, 1996	2.23	2.30	0.07	0.03	8	Age, education, BMI, age at menarche, age at 1st live birth, oral contraceptives, FHBC, benign breast diseases
Rollison DE, 2008	0.82	0.80	-0.02	-0.02	9	Age, menopausal status, BMI, age at menarche, age at first live birth , oral contraceptive use , other hormone use , FHBC , alcohol consumption
Smith SJ, 1994	1.63	1.58	-0.05	-0.03	10	Age (<32, 32+ years), region, age at menarche, nulliparity, age at first full-term pregnancy, breastfeeding, total oral contraceptive use, FHBC (mother or sister), biopsy for benign breast disease, alcohol consumption at age 18
Johnson KC, 2000	1.74	1.48	-0.26	-0.15	10	Age at 1st pregnancy (> 5 months), live births, breast feeding, BMI, height, alcohol, physical activity (2 years before interview)
Lash TL, Aschengrau A, 2002	0.89	0.85	-0.04	-0.04	10	Age, age at first pregnancy, parity, history of benign breast disease, BMI, FHBC, history of breast cancer, radiation therapy, benign breast diseases , alcohol
De Silva et al, 2010	2.55	2.90	0.35	0.14	12	Breastfeeding, reproductive factors, age at 1st pregnancy, age at menarche, menopause, past history of abortions, hormonal contraceptives use, age, BMI, highest education level, employed, FHBC
Lissowska J, 2006	1.09	1.11	0.02	0.02	13	Age, site, education, age at menarche, number of full-term births, age at first full-term birth, age at menopause, BMI, FHBC, prior benign biopsy, previous screening mammography, oral contraceptive use and use of hormone replacement therapy
Nishino Y, 2014	1.08	1.09	0.01	0.01	13	Age, BMI, occupation, physical activity, menopausal status, age at menarche, age at menopause, age at first birth, FHBC, parity number, use of exogenous female hormone or oral contraceptives, refer status, year of recruitment, area of residence and alcohol drinking

Study	Crude OR	Adjusted OR	Deviation	Relative deviation	Number of adjusted	Variable of adjusted
Pimhanam C, 2014	1.97	2.27	0.30	0.15	21	Age, religion, education, marital status, occupation body mass index; BMI, history of breast tissue biopsy menstrual status, regularity of menstrual cycle, age at menarche, number of full term pregnancies, age at first delivery, history of abortion in terms of number and age at first abortion, history of breast feeding, use of oral contraceptive pills; OCP first degree relatives with breast cancer exercise, alcohol and grilled food consumption.

* OR: Odds Ratio;

Deviation=crude OR-adjusted OR;

Relative Deviation (RD) = (crude OR-adjusted OR)/crude OR;

Order by number of adjusted variable;

FHBC: family history of breast cancer;

BMI: body mass index.
